# Supplementary material for: A new compression strategy to reduce the size of nanopore sequencing data
Source: Genome Res. 2025 Jul;35(7):1574–82. doi: 10.1101/gr.280090.124 (PMC12212073; doi:10.1101/gr.280090.124)
Supplement: Supplement 1 [file Supplemental_Materials_Combined.pdf]

LIST OF SUPPLEMENTAL MATERIALS

Supplemental Table S1. Summary of datasets used for evaluating compression methods **p2**

Supplemental Table S2. Definitions of lossless compression methods evaluated for compression of nanopore signal data **p3**

Supplemental Table S3. Use of computational resources for lossless compression methods **p4**

Supplemental Table S4: Impact of ex-zd bit-reduction on basecalling accuracy for the dataset 'HG002-Prom5K Chr22 subset' **p5**

Supplemental Table S5. Analysis of outlier reads in read-by-read basecalling analysis. **[Attached as separate Excel file]**

Supplemental Table S6: Impact of ex-zd bit-reduction on 5mC profiling for the dataset 'HG002-Prom5K Chr22 subset' **p6**

Supplemental Table S7: Effect of ex-zd bit-reduction on basecalling and 5mC profiling for the dataset 'HG002-Prom4K Chr22 subset' **p7**

Supplemental Table S8: Effect of ex-zd bit-reduction on basecalling and 5mC profiling for the dataset 'HG001-PromR9 Chr22 subset' **p8**

Supplemental Table S9: Effect of ex-zd bit-reduction on basecalling for the dataset 'HG002-Min5K' **p9**

Supplemental Table S10: Effect of ex-zd bit-reduction on basecalling for the dataset 'UHRR-Prom' and 'SIRV-Min' **p10**

Supplemental Note 1. Exploration of alternative compression methods on top of bit-reduced data **p11**

Supplemental Note 2. Mathematical comparison between the size of ex-zd and svb12-zd **p12**

Supplemental Note 3. Commands and versions used for experiments **p13**

Supplemental Figure S1. Supporting analyses for evaluation of ex-zd lossy compression **p16**

Supplemental Figure S2. Impact of GPU hardware on basecalling outcomes **p17**

Supplemental Figure S3. Evaluating ex-zd bit-reduction strategy for lossy compression of ONT MinION data **p18**

Supplemental Table S1. Summary of datasets used for evaluating compression methods. Note: Ex-zd 3-bit reduction refers to an 8-bit encoding for PromethION datasets (where native encoding is 11-bit) and 10-bit encoding for MinION datasets (where native encoding is 13-bit).

Dataset	Device	Kit	Pore	Data rate (kHz)	Reads (millions)	Ave read length (kb)	Total seq. (Gbases) / Genome cov.	FASTQ gzip (GiB)	POD5 VBZ (GiB)	BLOW5 VBZ lossless (GiB)	BLOW5 ex-zd, lossless (GiB)	BLOW5 ex-zd 3-bit reduction (GiB)
HG002-Prom5K	PromethION	LSK114	10.4.1	5	18.8	6.9	131 / 42x	119.2	1661	1659	1622	924
HG002-Prom5K (Chr22 subset)	PromethION	LSK114	10.4.1	5	0.2	6.9	0.9 / 42x	0.8	9.9	9.9	9.7	5.5
HG002-Prom4K	PromethION	LSK114	10.4.1	4	15.3	6.7	102 / 33x	96	1075	1073	1041	609
HG002-Prom4K (Chr22 subset)	PromethION	LSK114	10.4.1	4	0.2	6.7	1.6 / 33x	1.4	14.2	14.1	13.7	8.0
HG002-Min5K	MinION	LSK114	10.4.1	5	2.7	2.9	7.9 / 2.5x	7.5	113.7	113.6	111.6	63.5
UHRR-Prom	PromethION	RNA004	RP4	4	15.4	1.1	17.6 / NA	16.6	549	549	539	318
UHRR-Prom (500K read subset)	PromethION	RNA004	RP4	4	0.5	1.1	0.6 / NA	0.5	16.8	16.7	16.4	9.7
SIRV-Min	MinION	RNA004	RP4	4	0.03	0.99	.028 / NA	0.0211	0.6617	0.6608	0.6527	0.3792
HG001-PromR9 (Chr22 subset)	PromethION	LSK109	9.4.1	4	0.1	15	1.5 / 31x	1.2	11.1	11.1	11.1	6.0
UHRR-PromR9	PromethION	RNA002	9.4.1	3	1	1.2	1.2 / NA	1.2	60.4	60.3	60.1	33.1

Supplemental Table S2. Definitions of lossless compression methods evaluated for compression of nanopore signal data. See **Figure 1** for a comparison of compression ratios achieved by different methods and their combinations.

Comp. method	Description
^_*	apply * then ^
bzip2	bzip2 with compression level 9
ex-zd	see **Methods**
fast_lzma2	Fast LZMA2 with compression level 6
flac_P11	FLAC with 12 bits per sample, sampling rate 4000, compression level 5 and one channel
huffman	huffman on the one-byte data
none	no compression
shuffman	huffman but use a pre-built table
submin	subtract the minimum
svb	StreamVByte: integers are binned into 1,2,3 and 4 bytes
svb0124	svb but integers are binned into 0,1,2 and 4 bytes
svb12	svb but integers are binned into 1 and 2 bytes
uint	bitpacking
vb1e2	write the one-byte data followed by two-byte data
vbbe21	vbe21 but minimally bitpack the position deltas and data
vbe21	write the two-byte data followed by one-byte data
vbse21	vbe21 but svb encode the position deltas and minimally svb12 encode the two-byte data
zd	apply the zig-zag delta transformation
zlib	zlib with the default compression level
zsm	subtract the mean and apply zig-zag transformation
zstd	Zstandard with compression level 1

Supplemental Table S3. Use of computational resources for lossless compression methods.

Values indicate the time and memory required to decompress then re-compress a typical ONT dataset (HG002-Prom5K; see **Supplemental Table S1**). For each compression type, decompression and compression were performed together using slow5tools view with 40 threads, and the total execution time and the peak RAM usage were measured (the compression type of the input BLOW5 and the output BLOW5 was the same).

Compression Method	Time (hours)	Memory Usage (GiB)
zlib_svb_zd	4.15	5.12
zlib_ex-zd	3.81	4.56
zstd_svb_zd	2.97	4.85
zstd_ex-zd	2.90	4.47
zstd_svb12_zd (VBZ)	2.72	4.41

Supplemental Table S4: Impact of ex-zd bit-reduction on basecalling accuracy for the dataset 'HG002-Prom5K Chr22 subset' (see Supplemental Table S1). Accuracy is assessed by mean and median read-to-reference identity scores. Note: basecalling models for Dorado 0.3.4 and Guppy 6.5.7 were stated to be equivalent in documentation from ONT.

No. of bits	Dorado 0.3.4				Guppy 6.5.7			
	SUP		HAC		SUP		HAC	
	mean	median	mean	median	mean	median	mean	median
11 bit	0.9321	0.9839	0.9240	0.9751	0.9321	0.9839	0.9239	0.9749
10 bit	0.9321	0.9839	0.9240	0.9750	0.9320	0.9839	0.9238	0.9748
9 bit	0.9319	0.9837	0.9238	0.9748	0.9320	0.9838	0.9237	0.9746
8 bit	0.9314	0.9831	0.9231	0.9739	0.9314	0.9831	0.9229	0.9738
7 bit	0.9292	0.9805	0.9200	0.9701	0.9293	0.9806	0.9199	0.9699
6 bit	0.8965	0.9377	0.8740	0.9089	0.8963	0.9378	0.8733	0.9080
5 bit	0.7162	0.7159	0.7094	0.7024	0.7100	0.7031	0.7100	0.7031

Supplemental Table S6: Impact of ex-zd bit-reduction on 5mC profiling for the dataset 'HG002-Prom5K Chr22 subset' (see Supplemental Table S1). Methylation profiling assessed by Pearson correlation of 5mC frequencies at global CpG sites to matched wgBS data (on an HG002 reference sample). Note: basecalling models for Dorado 0.3.4 and Guppy 6.5.7 were stated to be equivalent in documentation from ONT. f5c was run using a model trained for 4KHz data, as a model for 5KHz is not yet available.

No. of bits	Dorado 0.3.4		Guppy 6.5.7		f5c
	SUP	HAC	SUP	HAC	4kHz model
11 bit	0.9264	0.9253	0.9282	0.9259	0.8707
10 bit	0.9263	0.9255	0.9281	0.9260	0.8707
9 bit	0.9265	0.9257	0.9280	0.9259	0.8703
8 bit	0.9261	0.9254	0.9275	0.9256	0.8699
7 bit	0.9266	0.9243	0.9267	0.9262	0.8690
6 bit	0.9159	0.9050	0.9177	0.9054	0.8431
5 bit	0.2842	0.4306	0.2399	0.3991	0.3053

Supplemental Table S7: Effect of ex-zd bit-reduction on basecalling and 5mC profiling for the dataset 'HG002-Prom4K Chr22 subset' (see Supplemental Table S1). 'Sequence' indicates mean read-to-reference identity scores. 'Methylation' indicates 5mC frequency correlation with matched wgBS data (HG002 reference sample), similar to **Supplemental Tables S4, S6.**

No. of bits	Dorado 0.3.4			
	SUP		HAC	
	Sequence	Methylation	Sequence	Methylation
11 bit	0.9042	0.9433	0.8961	0.9425
8 bit	0.9037	0.9435	0.8955	0.9424

Supplemental Table S8: Effect of ex-zd bit-reduction on basecalling and 5mC profiling for the dataset 'HG001-PromR9 Chr22 subset' (see Supplemental Table S1). 'Sequence' indicates mean read-to-reference identity scores. 'Methylation' indicates 5mC frequency correlation with matched wgBS data (HG001 reference sample), similar to **Tables S4, S6.**

No. of bits	Dorado 0.3.4			
	SUP		HAC	
	Sequence	Methylation	Sequence	Methylation
11 bit	0.9105	0.9091	0.8968	0.9049
8 bit	0.9010	0.8944	0.8853	0.8836

Supplemental Table S9: Effect of ex-zd bit-reduction on basecalling for the dataset 'HG002-Min5K' (see Supplemental Table S1). Values indicate mean read-to-reference identity scores.

No. of bits	Dorado 0.3.4	
	SUP	HAC
13 bit	0.9456	0.9360
10 bit	0.9443	0.9342

Supplemental Table S10: Effect of ex-zd bit-reduction on basecalling for the dataset 'UHRR-Prom' and 'SIRV-Min' (see Supplemental Table S1). Values indicate mean read-to-reference identity scores.

No. of bits	Dorado server 7.2.13 (Dorado 0.4.0)<br><br>Human mRNA (Prom.)	
	SUP	HAC
11 bit	0.9579	0.9494
8 bit	0.9567	0.9480
**No. of bits**	**Dorado server 7.2.13 (Dorado 0.4.0)<br><br>Synthetic RNA (MinION)**	
	**SUP**	**HAC**
13 bit	0.9557	0.9328
10 bit	0.9529	0.9249

Supplemental Note 1. Exploration of alternative compression methods on top of bit-reduced data

Because bit-reduced signal data has a different structure from native ONT data, we reasoned that different compression methods might exhibit different performances when applied after the bit-reduction process described in our study. To test this, we re-ran each of the lossless compression methods evaluated in **Figure 1** (n = 44) on the same dataset (HG002-Prom5K Chr22 subset) but encoded with different numbers of bits; ranging from native 11-bit encoding down to 7-bit encoding. We re-calculated the compression ratio for each combination of bit-reduction and lossless compression methods and these are plotted below.

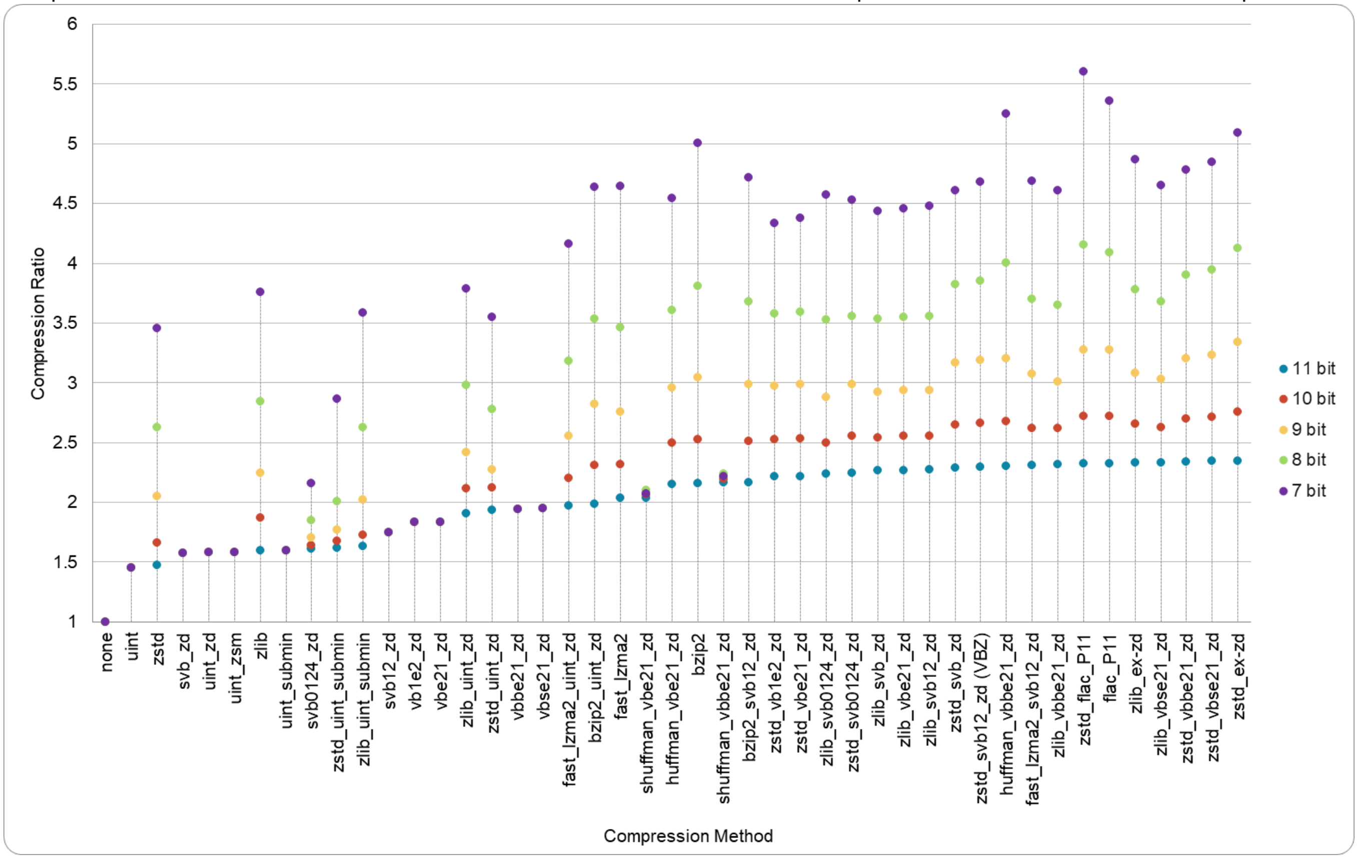

The methods in the plot above are presented in ascending order of the compression ratios for lossless compression (11-bit PromethION data). The top-performing compression algorithms for 11-bit lossless data achieve only slightly better compression ratios than others because lossless compression is nearing its theoretical limits, efficiently capturing all information content and reducing redundancy. However, we see relatively large differences between methods on bit-reduced (e.g. 7-bit) encodings. Particularly, the order of the compression ratios for 11-bit native data does not hold when applied to bit-reduced data. For instance, on 7-bit data (4-bits reduced) zstd_flac_P11 (which uses Free Lossless Audio Codec (FLAC) designed for audio compression) was the best performer. Algorithms based on the DEFLATE framework (which combines Huffman coding and LZ77 compression) like zlib and zstd also tend to perform well on bit-reduced data, likely due to their use of prefix codes being optimal on bit-reduced raw signal data. Applying DEFLATE techniques on top of zigzag delta encoding (which encodes and stores the differences between consecutive data points in the data series), performs further well with lossy bit manipulation. This is because the differences are more repetitive due to the bit-reduction.

This analysis shows that different compression strategies may be preferable for the compression of bit-reduced data, compared to those for compression of native 11-bit data. While some lossless methods do not provide better compression ratios for bit-reduced data than the original unaltered data, removing more bits improves the compression ratio for most methods. Overall, this analysis indicates there are significant additional compression gains that may be obtained by applying alternative compression strategies, such as FLAC, on top of bit-reduced data. This subject warrants further exploration.

Supplemental Note 2. Mathematical comparison between the size of ex-zd and svb12-zd

When the number of exceptions is greater than one, the number of data points must be greater than 185 and the proportion of exceptions must be less than 0.028 for ex-zd to consume fewer bytes than svb12-zd. When there are no exceptions, the number of data points must be greater than 121; when there is only one exception, the number of data points must be greater than 169. For a PromethION DNA LSK114 5KHz dataset (HG002-Prom5K; see **Table S1**) with ~22 million reads, the mean proportion of exceptions per read was found to be 0.020; the mean number of data points per read 93936; and according to these mathematical propositions ex-zd consumes fewer bytes than svb12-zd 89.2% of the time.

Let $L_z: N \times N \rightarrow N$ be the length in bytes of an encoding z given the number of integers n and the number of two-byte exceptions n_x. Then, the length of svb12-zd is given by:

$$L_{svb12\text{-}zd}(n, n_x) = ceil(n/8) + n + n_x$$

For ex-zd there are three cases:

1. $n_x = 0$: $L_{ex\text{-}zd}(n) = 16 + n$
2. $n_x = 1$: $L_{ex\text{-}zd}(n) = 23 + n$
3. $n_x > 1$: $L_{ex\text{-}zd}(n, n_x) <= 23 + 2\ ceil(n_x/4) + 5n_x + n$

Case 3 is a worst case upper bound which occurs when all the exceptions' positions' deltas are in the range $[2^{24}, 2^{32})$. For cases 1 and 2, $L_{ex\text{-}zd}$ is smaller than $L_{svb12\text{-}zd}$ when $n > 121$ and $n > 169$ respectively. For case 3:

- $23 + 2\ ceil(n_x/4) + 5n_x + n < ceil(n/8) + n + n_x$
- $23 + n_x/2 + 5n_x + n < (n + 7)/8 + n + n_x$
- $n_x < (n - 177)/36$

which implies that (for $n_x > 1$) $L_{ex\text{-}zd}$ is smaller than $L_{svb12\text{-}zd}$ when $n > 177$ and $n_x/n < 0.028$ (as n tends to infinity). In practice, for PromethION DNA data sequenced on kit LSK114 the expected number of integers is $E[N] = 93936.3$, the expected number of exceptions is $E[N_x] = 1746.41$ and the expected proportion of exceptions is $E[N_x/N] = 0.020$, which satisfies the conditions for $L_{ex\text{-}zd} < L_{svb12\text{-}zd}$ and give the following expected space saving per read:

$$E[L_{svb12\text{-}zd} - L_{ex\text{-}zd}] >= E[(N - 177)/36 - N_x] > 858 \text{ bytes}$$

Note: these calculations are irrespective of the application of Zstandard.

Supplemental Note 3. Commands and versions used for experiments

Size and Performance measurements

```
# lossless conversion to the compression type ${REC_MTD}_${SIG_MTD} (e.g., REC_MTD=zstd, SIG_MTD=ex-zd)
slow5tools view reads.blow5 -c ${REC_MTD} -s ${SIG_MTD} -o reads_${REC_MTD}_${SIG_MTD}.blow5 -t40

# converting to POD5
blue-crab s2p reads.blow5 -o reads.pod5 -p 40

# lossy conversion eliminating $COUNT bits
slow5tools degrade reads.blow5 -c ${REC_MTD} -s ex-zd -b $COUNT -o reads_${REC_MTD}_ex-zd_${COUNT}.blow5 -t40

# measure size in bytes
du -b <file>

# measure time and RAM for decompressing and compressing to the same compression type
clean_fscache #see https://github.com/hasindu2008/biorand/blob/master/clean_fscache.c
/usr/bin/time -v slow5tools view reads_${REC_MTD}_${SIG_MTD}.blow5 -c ${REC_MTD} -s ${SIG_MTD} -o reads_tmp.blow5 -t40
```

Versions:

- for zlib+svb-zd, zlib+ex-zd, zstd+svb-zd and zstd+ex-zd: slow5tools 1.3.0
- for zstd+svb12-zd: slow5tools vbz branch [https://github.com/hasindu2008/slow5tools/tree/vbz commit 8a366bf6dffe0c94fd0ec148cca22f09e47c31e5]. Note: please use *-s svb16-zd* instead of *-s svb12-zd* (same thing, different naming conventions)
- blue-crab 0.1.0

Accuracy Evaluation

Lossy compression

```
sigtk qts -b $COUNT --method=round reads_chr22.blow5 -o rounded_$COUNT.blow5
# COUNT: 0, 1, 2, etc where it is the number of bits eliminated
```

The data generated from the above command will be identical to that from the following slow5tools command:

```
slow5tools degrade -b $COUNT reads_chr22.blow5 -o rounded_$COUNT.blow5 -c zlib -s svb-zd
```

Versions: sigtk 0.2.0, slow5tools 1.3.0

DNA basecalling

```
# Guppy via buttery-eel
buttery-eel -g ont-guppy/bin/ -x cuda:all --port 5000 --config <model_guppy> -i reads_chr22.blow5 -o reads_chr22.fastq

# Dorado through slow5-dorado
slow5-dorado basecaller <model_dorado> --emit-fastq reads_chr22.blow5 > reads_chr22.fastq

# alignment
minimap2 -ax map-ont hg38noAlt.fa --secondary=no reads.fastq -o reads.sam

# identity scores
samtools view -F 2308 -h reads.sam -o reads_primary.sam
paftools.js sam2paf reads_primary.sam | awk '{print $1"\t"$10/$11} > primary_identity_scores.txt
```

Flowcell	sampling_rate	model type	model_guppy	model_dorado
R10.4.1	5 kHz	super accuracy	dna_r10.4.1_e8.2_400bps_5khz_sup.cfg	dna_r10.4.1_e8.2_400bps_sup@v4.2.0
		high accuracy	dna_r10.4.1_e8.2_400bps_5khz_hac_prom.cfg	dna_r10.4.1_e8.2_400bps_hac@v4.2.0
	4 kHz	super accuracy	dna_r10.4.1_e8.2_400bps_sup.cfg	dna_r10.4.1_e8.2_400bps_sup@v4.1.0
		high accuracy	dna_r10.4.1_e8.2_400bps_hac_prom.cfg	dna_r10.4.1_e8.2_400bps_hac@v4.1.0
R9.4.1	4 KHz	super accuracy	dna_r9.4.1_450bps_sup_prom.cfg	dna_r9.4.1_e8_sup@v3.3
		high accuracy	dna_r9.4.1_450bps_hac_prom.cfg	dna_r9.4.1_e8_hac@v3.3

Versions: buttery-eel 0.4.1 through Guppy 6.5.7, slow5-dorado 0.3.4, minimap 2.26

DNA variant calling

```
# sort the alignments
samtools sort reads_primary.sam -o reads.bam
samtools index reads.bam

# variant calling
run_clair3.sh --bam_fn=reads.bam --ref_fn=hg38noAlt.fa --threads=32 --include_all_ctgs --platform="ont" --
model_path=<model_clair3> –output=clair3_out/ --enable_phasing --longphase_for_phasing --sample_name=reads

# variant evaluation
rtg vcfeval -b HG002_GRCh38_1_22_v4.2.1_benchmark.vcf.gz -c clair3_out/merge_output.vcf.gz  -o out_rtg/ -t
hg38noAlt.sdf --region chr22:1-50818468 -e HG002_GRCh38_1_22_v4.2.1_benchmark_noinconsistent.bed --vcf-score-field QUAL
rtg rocplot out_rtg/snp_roc.tsv.g
```

Flowcell	sampling_rate	model type	model_clair3
R10.4.1	5 kHz	super accuracy	r1041_e82_400bps_sup_v420
		high accuracy	r1041_e82_400bps_hac_v420

Versions: clair3 1.0.10, rtg 3.12.1

DNA 5mC methylation calling

```
# Guppy Remora
buttery-eel -g ont-guppy/bin/ -x cuda:all --port 5000 --config <model_guppy> --call_mods -i reads_chr22.blow5 -o
reads_chr22.sam

# Dorado Remora
slow5-dorado basecaller <model_dorado>  --modified-bases "5mCG_5hmCG" reads_chr22.blow5 > reads_chr22.sam

# meth frequency for Remora
samtools fastq -TMM,ML reads_chr22.sam |  minimap2 -x map-ont -a -y --secondary=no hg38noAlt.fa  - | samtools sort -@32
- > reads_chr22_mapped.bam
samtools index reads_chr22_mapped.bam
modkit pileup --cpg --ref hg38noAlt.fa --ignore h reads_chr22_mapped.bam reads_remora.bedmethyl
grep "chr22" reads_remora.bedmethyl | grep -v nan  > reads_remora_chr22.bedmethyl

# f5c
f5c call-methylation -x hpc-low -g hg38noAlt.fa -b reads_chr22_mapped.bam -r reads_chr22.fastq -w chr22 --slow5
reads_chr22.blow -o reads_chr22.tsv
f5c meth-freq -s -i reads_chr22.tsv -o freq_chr22.tsv

# getting correlation and plots  based on compare_methylation.py and plot_methylation.R are available in f5c repository
```

```
python3 compare_methylation.py chr22_bi.tsv reads_remora_chr22.bedmethyl > bi_vs_remora.tsv
R --no-save --args bi_vs_remora.tsv < plot_methylation.R

# getting individual modification calls
modkit extract reads_chr22_mapped.bam mods.tsv
```

Flowcell	sampling_rate	model type	model_guppy	model_dorado
R10.4.1	5 kHz	super accuracy	dna_r10.4.1_e8.2_400bps_5khz_modbases_5mc_cg_sup_prom.cfg	dna_r10.4.1_e8.2_400bps_sup@v4.2.0
		high accuracy	dna_r10.4.1_e8.2_400bps_5khz_modbases_5mc_cg_hac_prom.cfg	dna_r10.4.1_e8.2_400bps_hac@v4.2.0
	4 kHz	super accuracy	dna_r10.4.1_e8.2_400bps_modbases_5mc_cg_sup_prom.cfg	dna_r10.4.1_e8.2_400bps_sup@v4.1.0
		high accuracy	dna_r10.4.1_e8.2_400bps_modbases_5mc_cg_hac_prom.cfg	dna_r10.4.1_e8.2_400bps_hac@v4.1.0
R9.4.1	4 KHz	super accuracy	dna_r9.4.1_450bps_modbases_5mc_cg_sup_prom.cfg	dna_r9.4.1_e8_sup@v3.3
		high accuracy	dna_r9.4.1_450bps_modbases_5mc_cg_hac_prom.cfg	dna_r9.4.1_e8_hac@v3.3

Versions: buttery-eel 0.4.1 through Guppy 6.5.7, slow5-dorado 0.3.4, f5c 1.4, minimap 2.26, modkit 0.1.13

RNA basecalling

```
# Dorado server via buttery-eel
buttery-eel -g ont-dorado-server/bin/ -x cuda:all --port 5000 --config <model> -i reads_500k.blow5  -o reads_500k.fastq

# alignment
minimap2 -ax map-ont -uf --secondary=no gencode.v40.transcripts.fa/ spike-in_transcripts.fa reads.fastq -o reads.sam

# identity scores
samtools view -F 2308 -h reads.sam -o reads_primary.sam
paftools.js sam2paf reads_primary.sam | awk '{print $1"\t"$10/$11} > primary_identity_scores.txt
```

Flowcell	sampling_rate	model type	model
RP4	4 kHz	super accuracy	rna_rp4_130bps_sup.cfg
		high accuracy	rna_rp4_130bps_hac_prom.cfg
R9.4.1	3 kHz	high accuracy	rna_r9.4.1_70bps_hac_prom.cfg

Versions: buttery-eel 0.4.2 through ont-dorado-server 7.2.13 (equivalent to Dorado 0.4.0), minimap 2.26

RNA 6mA methylation calling

```
samtools sort reads_primary.sam -o reads.bam
samtools index reads.bam

f5c index reads.fastq --slow5 reads_500k.blow5
f5c eventalign --reads reads.fastq --bam reads.bam -g gencode.v40.transcripts.fa --slow5 reads_500k.blow5 --scale-
events --signal-index --summary f5c_summary.tsv -o f5c.tsv --rna --kmer-model rna004.nucleotide.5mer.model --min-mapq 0

m6anet dataprep --eventalign f5c.tsv --out_dir m6anet/ --n_processes 8
m6anet inference --input_dir m6anet/ --out_dir m6anet/ --pretrained_model HEK293T_RNA004 --n_processes 8 --
num_iterations 1000
```

Versions: f5c 1.4, m6anet 2.1.0

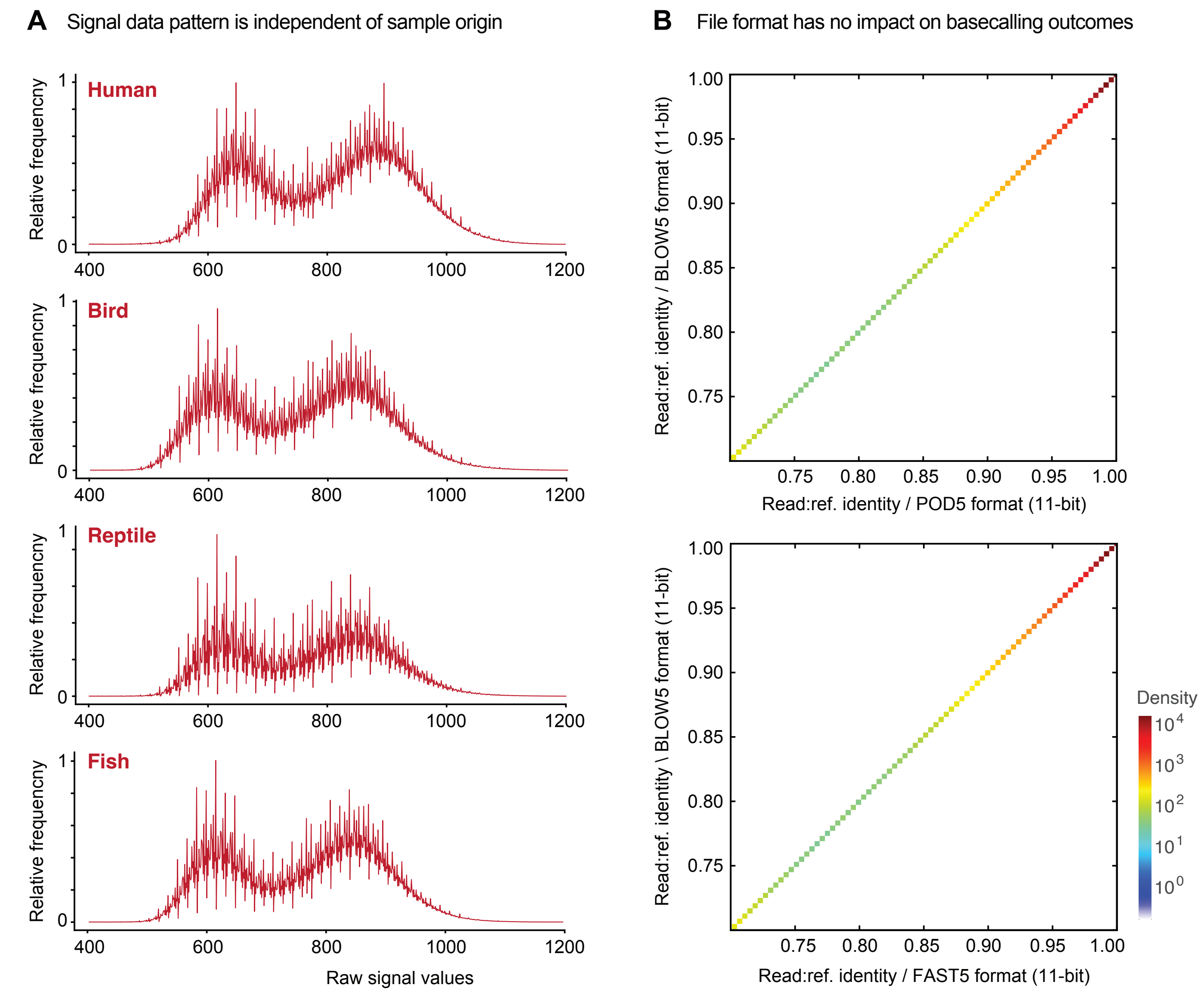

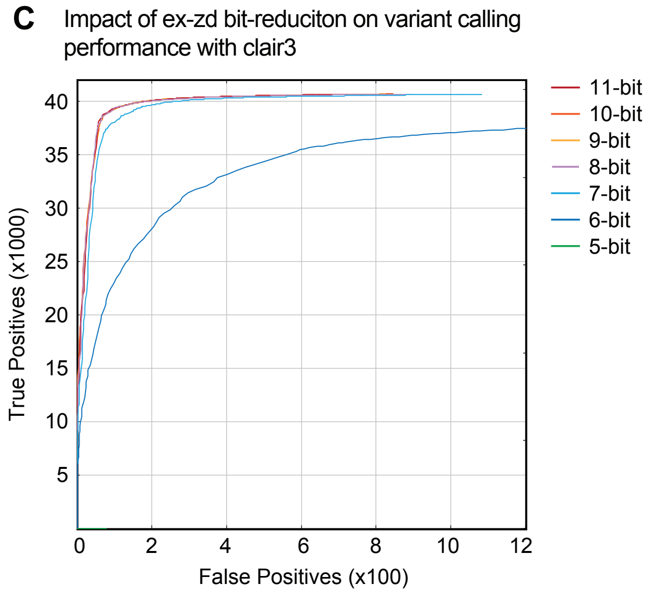

Supplemental Figure S1. Supporting analyses for evaluation of ex-zd lossy compression. (**A**) Frequency distributions for raw signal values in ONT PromethION datasets generated from human genomic DNA (top), as well as DNA from a representative bird, reptile and fish species, represented in native 11-bit encoding (red). The characteristic 'spiked' frequency pattern is observed regardless of species, and has no relationship to the k-mer frequency profile in a given sample. (**B**) Density scatter plots show read:reference identities for individual basecalled reads. The left plot compares 11-bit data in BLOW5 format vs native POD5 format, both basecalled with Guppy SUP model. The right plot compares 11-bit data in BLOW5 format vs native FAST5 format, both basecalled with Guppy SUP model. These plots confirm that when no lossy compression is applied, the choice of file format (either POD5, FAST5 or BLOW5) has no impact on base calling outcomes. (**C**) ROC curves show the accuracy of germline variant calling using Clair3 for the HG002 genome reference sample (HG002-Prom5k chr22 subset; see **Supplemental Table S1**) represented in native 11-bit encoding (red) or encoded with a smaller number of bits (10–5 bits). The analysis shows ex-zd lossy compression has no impact on variant detection at an 8-bit encoding or above, echoing the basecalling analysis in **Figure 2**.

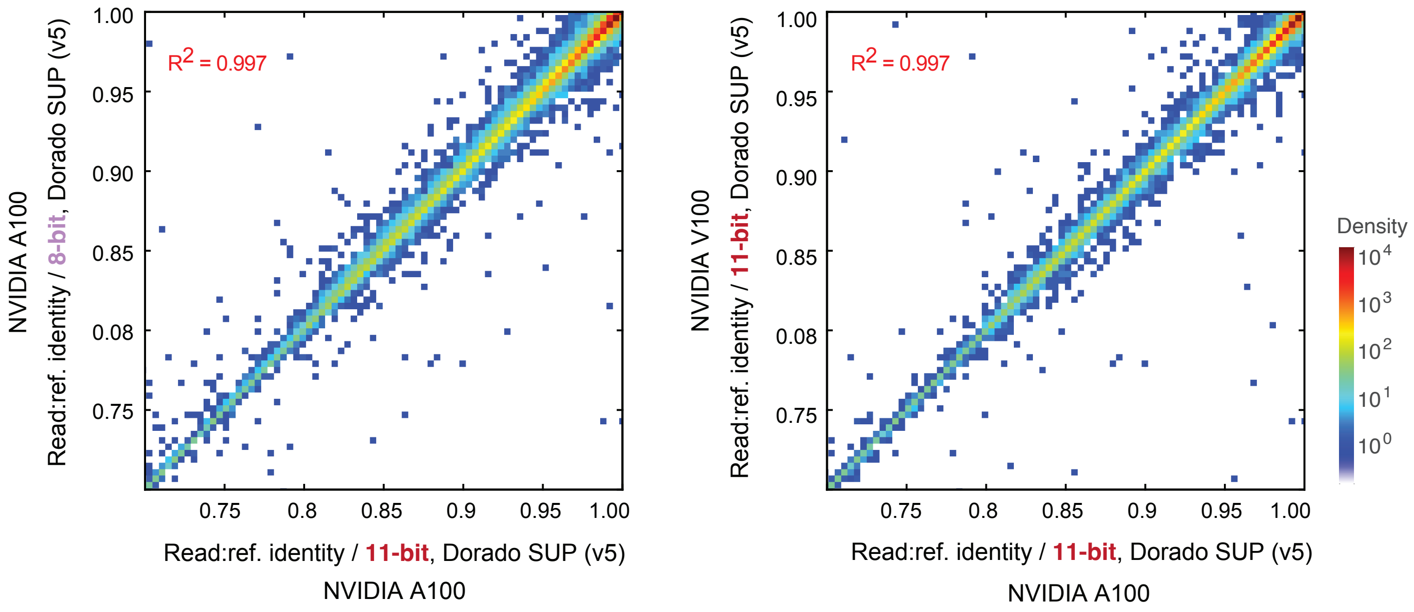

Supplemental Figure S2. Impact of GPU hardware on basecalling outcomes. Density scatter plots show read:reference identities for individual basecalled reads. The left plot compares 11-bit vs 8-bit data basecalled with the latest Dorado v5 SUP models on an NVIDIA A100 GPU. The right plot compares identical 11-bit data basecalled with the same v5 SUP models and Dorado software version but executed on either a NVIDIA V100 vs A100 GPU. The degree of scatter is equivalent between the 11-bit vs 8-bit comparison as identical data basecalled on different hardware.

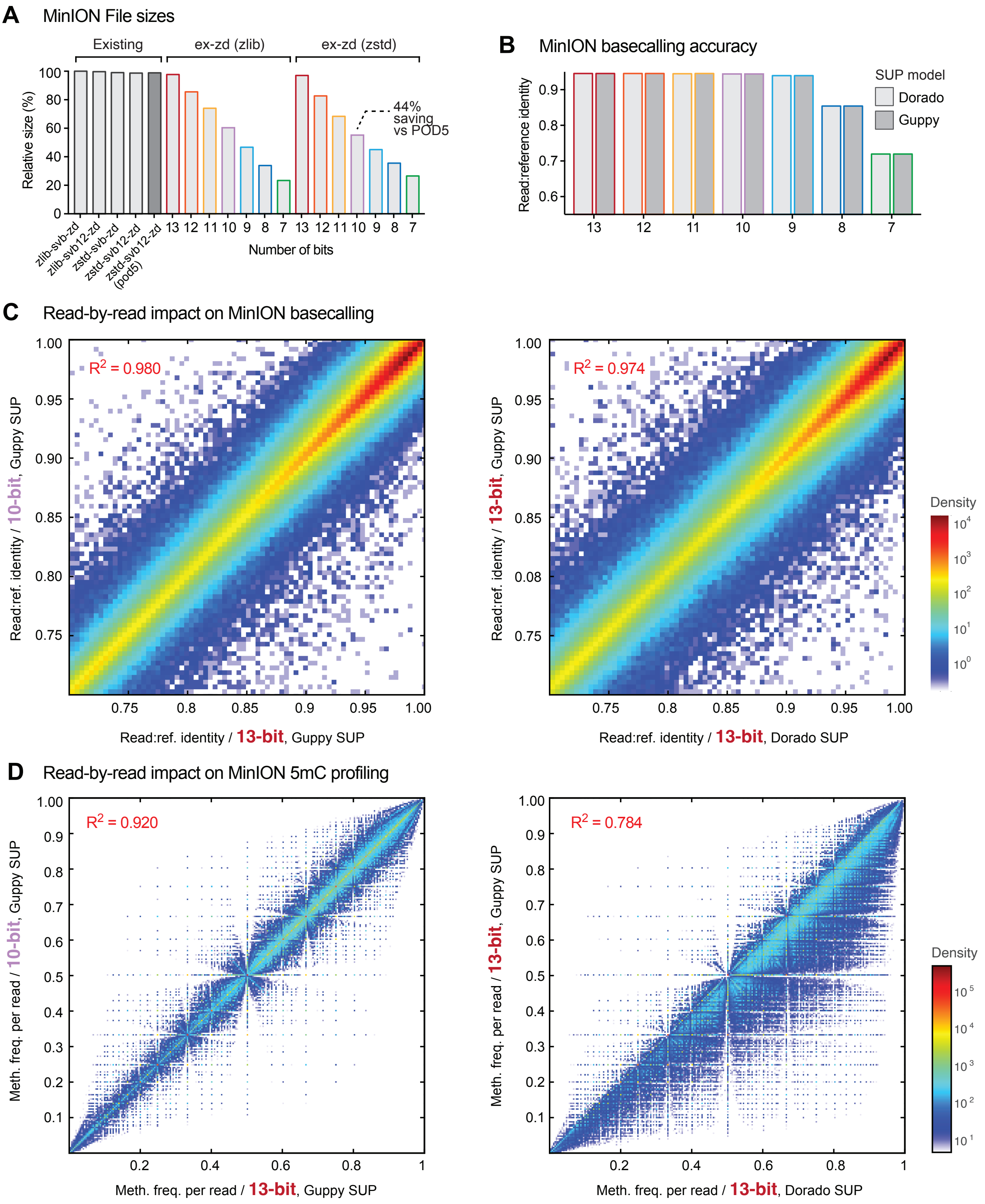

Supplemental Figure S3. Evaluating ex-zd bit-reduction strategy for lossy compression of ONT MinION data. (**A**) Bar chart shows relative file sizes for a typical ONT MinION dataset (HG002-Min5K; see **Supplemental Table S1**) with current lossless compression methods (grey bars) compared to lossy ex-zd compression with data encoded with a decreasing number of bits (native 13-bit down to 7-bit). Sizes are shown as percentages relative to zlib-svb-zd, which is currently the default compression method used in slow5tools/slow5lib. Native POD5 format, which uses zstd-svb12-zd compression, is shown for comparison. (**B**) Bar chart shows basecalling accuracy, as measured by mean read:reference identity, for the same dataset and bit-reduced encodings as above. Basecalling accuracies are shown separately for ONT's Dorado (light grey) vs Guppy (dark grey) software, both with SUP models. (**C**) Density scatter plots show read:reference identities for individual basecalled reads from the same dataset as above. The left plot compares native 13-bit data vs bit-reduced 10-bit data, both basecalled with Guppy SUP model. The right plot shows native 13-bit data basecalled with Guppy vs Dorado software, using a matched SUP basecalling model. (**D**) Density scatter plots show 5mC methylation frequencies for individual basecalled reads; i.e. the fraction of CpG bases within a given read that are called as being methylated. The left plot compares native 11-bit data vs bit-reduced 8-bit data, both basecalled with Guppy SUP model. The right plot shows native 11-bit data basecalled with Guppy vs Dorado software, using a matched SUP basecalling model.
